# Volcano-like intermittent bleeding activity for seven years from an arterio-enteric fistula on a kidney graft site after pancreas-kidney transplantation: a case report

**DOI:** 10.1186/1752-1947-4-357

**Published:** 2010-11-08

**Authors:** Peter Härle, Stephan Schwarz, Julia Langgartner, Jürgen Schölmerich, Gerhard Rogler

**Affiliations:** 1 Klinik für Rheumatologie und Physikalische Therapie, Katholisches Klinikum Mainz, An der Goldgrube 11, D-55131 Mainz, Germany; 2 Institute of Pathology, University of Regensburg, Franz-Josef-Strauss-Allee 11, Regensburg, D-93042, Germany; 3 Department of Internal Medicine I, University of Regensburg, Franz-Josef-Strauss-Allee 11, Regensburg, D-93042, Germany; 4 Department of Internal Medicine, University of Zürich, Rämistrasse 100, CH-8091 Zürich, Switzerland

## Abstract

**Introduction:**

We report the first case of a patient who underwent simultaneous kidney and pancreas transplantation and who then suffered from repeated episodes of severe gastrointestinal bleeding over a period of seven years. Locating the site of gastrointestinal bleeding is a challenging task. This case illustrates that detection of an arterio-enteric fistula can be very difficult, especially in technically-challenging situations such as cases of severe intra-abdominal adhesions. It is important to consider the possibility of arterio-enteric fistulas in cases of intermittent bleeding episodes, especially in transplant patients.

**Case presentation:**

A 40-year-old Caucasian man received a combined pancreas-kidney transplantation as a result of complications from diabetes mellitus type I. Thereafter, he suffered from intermittent clinically-relevant episodes of gastrointestinal bleeding. Repeat endoscopic, surgical, scintigraphic, and angiographic investigations during his episodes of acute bleeding could not locate the bleeding site. He finally died in hemorrhagic shock due to arterio-enteric bleeding at the kidney graft site, which was diagnosed post-mortem.

**Conclusions:**

In accordance with the literature, we suggest considering the removal of any rejected transplant organs in situations where arterio-enteric fistulas seem likely but cannot be excluded by repeat conventional or computed tomography-angiographic methods. Arterio-enteric fistulas may intermittently bleed over many years.

## Introduction

We report the case of a 40-year-old Caucasian man who had undergone simultaneous kidney and pancreas transplantation and who suffered from repeated severe gastrointestinal bleeding episodes over a period of seven years. Locating a gastrointestinal bleeding site is a challenging task. It is important to consider the possibility of arterio-enteric fistulas in cases of intermittent bleeding episodes, especially in transplant patients. To the best of our knowledge, it has not been previously described in the literature that an arterio-enteric fistula can intermittently be active over seven years and not be detected despite repeated and intense conservative and surgical diagnostic procedures.

## Case presentation

A 40-year-old Caucasian man was referred to our intensive care unit for further diagnostic work-up because of gastrointestinal bleeding of unknown location. After blood transfusions in the referring hospital, he presented with a hemoglobin level of 12.3 mg/dL at 3:45 pm.

In March 1997, he received a simultaneous pancreatic-duodenal transplantation connected to the right iliac artery and renal transplantation connected to the left iliac artery on the basis of long-standing diabetes mellitus type I. The transplantation procedure was more difficult due to abdominal adhesions caused by peritoneal dialysis over five years with recurrent bacterial peritonitis. Two episodes of hemoglobin-relevant bleeding occurred; the first five days after the transplantation and the second 14 days after. These were followed by surgical revisions of the severe adhesive abdomen without finding the bleeding site. In April 1998, July 1998, February 1999, and August 1999 acute and hemoglobin-relevant gastrointestinal bleeding episodes occurred. Repeated gastroscopy and colonoscopy, in addition to conventional and magnetic resonance (MR)-angiographies, and repeat exploratory surgeries with intra-operative endoscopies in cooperation with skilled endoscopists and Tc-erythrocyte scintigraphies, could not reveal the location of the bleeding. The renal graft lost function due to rejection in August 1998. In June 1999, he received a second renal graft on his left side, leaving the first kidney graft in place. The second renal graft also lost function due to rejection in April 2003 and hemodialysis was started in October 2003. The pancreas graft lost function in 2002 due to rejection.

At about 10 pm on the day of his admission to our unit, he complained of severe, colic-like diffuse abdominal pain. An ultrasound did not show cholelithiasis, kidney or bladder problems and an X-ray of the chest and abdomen did not show any air-fluid levels. Administration of butyl-scopalamine relieved the colic-like pain completely. At 2 am, in a routine blood-gas check, his hemoglobin was down to 7.9 mg/dL and two units of blood were transfused with adequate rise to 9.4 mg/dL after one unit of blood. At 5 am, he again complained of severe colic-like diffuse abdominal pain with nausea, tachycardia, and hypotension. His hemoglobin levels dropped to 5.7 mg/dL without showing bloody stools. Intravenous fluids, blood transfusions and catecholamines were administered immediately. Suddenly, he vomited massive amounts of blood mixed with large blood clots, making intubation impossible. He died of hemorrhagic shock.

**Figure 1:**
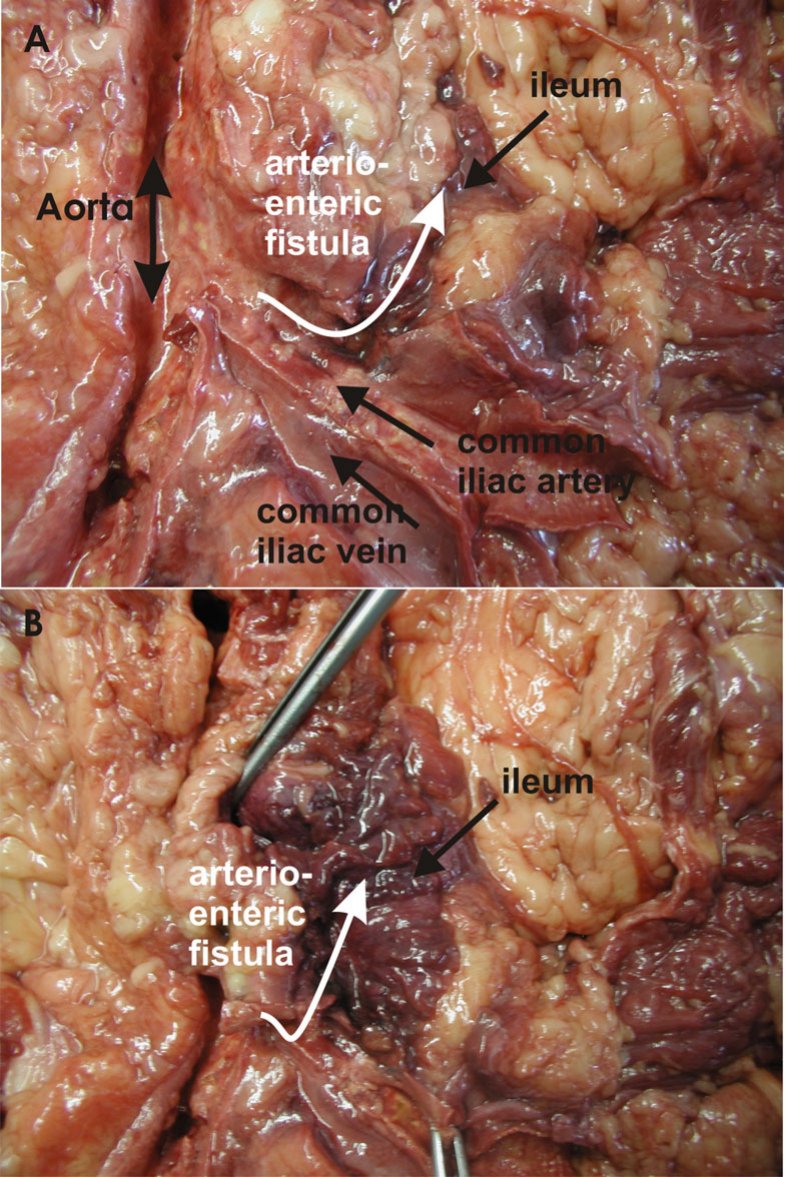
**(A) Anatomic situation of the aorta with left common iliac vein, artery, and arterio-enteric fistula to the ileum**. (B) Bloody residues are seen in the lumen of the ileum with fistula to the left common iliac artery.

Autopsy revealed extensive intra-abdominal adhesions. Meticulous exploration by the pathologist finally revealed an arterio-enteric fistula between his left common iliac artery, where the initial kidney was engrafted, and the adjacent ileum (Figure 1[A, B]). In addition, large blood clots were found distal to the fistula in his small intestine which led to intestinal obstruction; explaining the eruptive vomiting of blood instead of showing bloody stools. The obstruction with intestinal distension might also explain the colic-like pain [1] which was alleviated after administration of butyl-scopalamine.

## Discussion

Significant bleeding from an arterio-enteric fistula after pancreas transplantation is rare and associated with a high mortality rate [2]. In the literature, bleeding episodes are described in the setting of the context of pancreatitis of the transplanted organ and rejection reactions [1,3,4]. These inflammatory processes in close proximity to arterial vessels and the gut are likely to present the driving forces for the development of arterio-enteric fistula. Occurrences of arterio-enteric fistulas have also been described in other settings such as following pelvic radiation [5], aorto-iliac operations [6-8], biliary wallstent implantation [9], gastrointestinal and graft infections [10-12], spontaneously [6], and in chronic inflammatory bowel disease [13]. Emergency angiography with endovascular repair seems to be effective in controlling the acute bleeding situation [8,14,15]. However, a high rate of rebleeding is described and surgical removal of the transplanted pancreas showed the best survival outcome in the cases presented in the literature [1,2]. We describe for the first time that an arterio-enteric fistula can be intermittently active over seven years and not be detected despite repeated and intense conservative and surgical diagnostic procedures. Astonishingly, our case report stabilized after each acute bleeding episode, probably due to thrombotic occlusion of the fistula, making it impossible to detect it by surgery, endoscopic, angiographic, and scintigraphic methods. In our case report, the first bleeding episode occurred five days after his initial simultaneous pancreas-kidney transplantation. Rejection or pancreatitis as the cause of the fistula development was unlikely to have played a role during the first bleeding episode, as described in the above-mentioned transplant literature cases. Finally, it should be considered in our case report that there were severe abdominal adhesions caused by multiple bacterial peritonitis episodes during peritoneal dialysis prior to his first transplantation, thus enhancing the chance for surgical complications. In the follow-up period, the intra-abdominal adhesions were becoming increasingly problematic, giving the surgeons, the endoscopists, and finally the pathologist a challenge when inspecting our patient's intestine and organ graft sites.

## Conclusions

Retrospectively, we think that in renal and pancreatic transplant patients with gastrointestinal bleeding of obscure origin, even some years after transplantation after years, there should be a high suspicion for arterio-enteric fistulas. Therefore, we think that for these patients conventional- or computed tomography (CT)-angiography of the vascular insertion regions needs to be strongly suggested, repeatedly if necessary, to find the source of this bleeding [16,17].

However, in the case of inconclusive severe gastrointestinal bleeding, despite repetitive conventional or CT-angiographic examinations, it might be worth considering the removal of a rejected kidney along with the connecting vessels because arterio-enteric fistulas may be very difficult or even impossible to detect despite using the whole arsenal of medical diagnostics [18].

## Competing interests

The authors declare that they have no competing interests.

## Authors' contributions

PH wrote the manuscript. SS performed the pathological analysis and sectioning. JL, JS and GR, the attending physicians taking care of the patient on the intensive care unit, revised the manuscript. All authors read and approved the final manuscript.

## Consent

Written informed consent was obtained from the patient's next-of-kin for publication of this case report and any accompanying images. A copy of the written consent is available for review by the Editor-in-Chief of this journal.
